# Assessing the efficacy of mesotherapy products: Ultra Exo Booster, and Ultra S Line Plus in hair growth: An ex vivo study

**DOI:** 10.1111/srt.13780

**Published:** 2024-07-19

**Authors:** Shu Yi Zhou, Nguyen Ngan Giang, Hyunjee Kim, Pham Ngoc Chien, Linh Thi Thuy Le, Thuy‐Tien Thi Trinh, Pham Thi Nga, Han Jin Kwon, Jung Ryul Ham, Won Ku Lee, Yeon Ju Gu, Xin Rui Zhang, Yong Xun Jin, Sun Young Nam, Chan Yeong Heo

**Affiliations:** ^1^ Department of Medicine College of Medicine Seoul National University Seoul South Korea; ^2^ Department of Plastic and Reconstructive Surgery Seoul National University Bundang Hospital Seongnam South Korea; ^3^ Department of Medical Device Development College of Medicine Seoul National University Seoul South Korea; ^4^ Korean Institute of Nonclinical Study H&Bio. Co. Ltd. Seongnam South Korea; ^5^ Department of Biomedical Science College of Medicine Seoul National University Seoul South Korea; ^6^ Faculty of Medical Technique Hai Phong University of Medicine and Pharmacy Haiphong Vietnam; ^7^ UltraV Co., Ltd. R&D Center Seoul South Korea

**Keywords:** ex vivo, hairgrowth, hairloss, mesotherapy

## Abstract

In this study, scalp tissues from Korean adults between 20 and 80 without skin disease were used. Scalp tissues were processed, and hair follicles were isolated and cultured with different treatments (including Bioscalp, Ultra Exo Booster, and Ultra S Line Plus) from Ultra V company. Over 12 days, observations and measurements of hair follicle characteristics were recorded at intervals (Days 0, 3, 6, 9, and 12). The study assessed the impact of these substances on hair follicle growth and morphology. Bioscalp, combined with Ultra Exo Booster and Ultra S Line Plus, showed significant hair elongation in ex vivo. Preservation of hair bulb diameter was observed, indicating potential for sustained hair growth by exosome‐based products. The hair growth cycle analysis suggested a lower transition to the catagen stage in test products from Ultra V compared to non‐treated groups. The research findings indicated that the tested formulations, especially the combination of Bioscalp, Ultra Exo Booster, and Ultra S Line Plus, demonstrated significant effectiveness in promoting hair growth, maintaining the integrity of the hair bulb, and reducing the transition to the catagen stage. The study suggests promising alternative treatments for hair loss, illustrating results that were as good as those of the conventional testing product groups.

## 1 INTRODUCTION

Alopecia or hair loss linked to heredity, aging, and other factors has been a notable issue affecting people worldwide, which enforced numerous medical and mental distress that influence the patient's quality of life.^1^, ^2^ As one ages, achieving and maintaining healthy hair is an essential problem since hair is considered one characteristic to identify a distinct individual. Research on effective medications for hair loss prevention and regeneration has been conducted to address this widespread concern as a growing emphasis.^3^, ^4^

Minoxidil, which the Food and Drug Administration approved, has been topically used as a potent product to maintain existing hairs and promote hair growth at a concentration of 2%–5%.^5^, ^6^ Though the activation of hair growth has not been elucidated, minoxidil is elaborated to activate potassium channels situated in the smooth muscles of peripheral arteries, which enables the reduction of the telogen phase duration and extension of the anagen phase, resulting in a gradual increase in both the diameter and length of hair follicles.^7^, ^8^ While minoxidil stands out as an effective conventional treatment that stimulates hair growth, there exist some disadvantages, such as cosmesis, pruritus (skin itching), desquamation (skin shedding), and hirsutism (over hair growth).^9^ Additionally, the effectiveness of minoxidil in aiding hair growth is not persistently permanent and requires prolonged treatment, which may lead to imperative expenses. Therefore, an alternatively competent approach to facilitate hair growth is urged to be innovative.

Nanocarrier technology has been found to possess extensive applications in the exploration and creation of drug‐delivery systems across diverse domains.^10^, ^11^ Exosome‐based technology, one of the nanocarriers approaches fostering cell signaling, has emerged as a promising therapeutic method for tissue regeneration.^12^, ^13^ Demonstrating potential effectiveness in addressing dermatological conditions such as wound healing, oxidative stress, and other diseases, it is expected to be a potent method for root treatment that prevents hair loss and stimulates hair elongation.^14^, ^15^

Alternative methods using natural compounds are also used to stimulate hair growth as they consistently promote blood flow.^16^, ^17^, ^18^ For example, *Houttuynia cordada* ^19^ and *Camellia sinensis* (black tea),^20^ conventional herbal medicinal plants rich in antioxidant and anti‐inflammatory properties from the Orient are extensively utilized as a constituent for treating alopecia. Besides, oxidative stress caused by fat cell accumulation could serve as the mechanism through which an unhealthy scalp harms the hair follicle cells, leading to hair thinner and hair fall.^21^, ^22^ Therefore, products that assist with diminishing fat accumulation could be considered a new approach to stimulating hair growth.

Beyond conventional methodologies, our study assesses novel formulations developed by the Ultra V company, which have been used in mesotherapy for skin regeneration, hair regeneration, and growth stimulation within an ex vivo research framework. Bioscalp, a commercial product from Ultra V assisting hair growth, which contains medicinal herbs such as polygonum multiflorum root extract, *Houttuynia cordata* extract, *Camellia sinensis* leaf extract, and so forth, was individually examined to evaluate the hair elongation ability. The integration of Bioscalp and Ultra Exo Booster, an exosome‐based product conventionally used in mesotherapy in hair growth activation, was also assessed. The combination exhibited a notable enhancement in hair length and a mitigation of hair root shrinkage after 12 days of culture. Furthermore, when combining Ultra Exo Booster with another product featuring horse chestnut extract and lecithin (a phospholipid that aids with eliminating excessive fat accumulation), Ultra S Line Plus similarly elaborated the efficacy in hair elongation and preservation of hair bulb integrity compared to the outcomes of the preceding experiment in the same period of time.

## 2 RESULTS

In this study, we investigated the efficacy of Bioscalp combined with different exo boosters in hair growth stimulation. In this paper, minoxidil, as a conventional product from two different companies, was assumed to be the control group to compare with the testing group to evaluate the efficacy of the combination. The experiments were conducted with three trials of objects from three donors: a 56‐year‐old female (test group‐trial 1), a 44‐year‐old female (test group‐trial 2), and a 71‐year‐old female (test group‐trial 3).

### 2.1 Hair elongation measurement analysis

**FIGURE 1:**
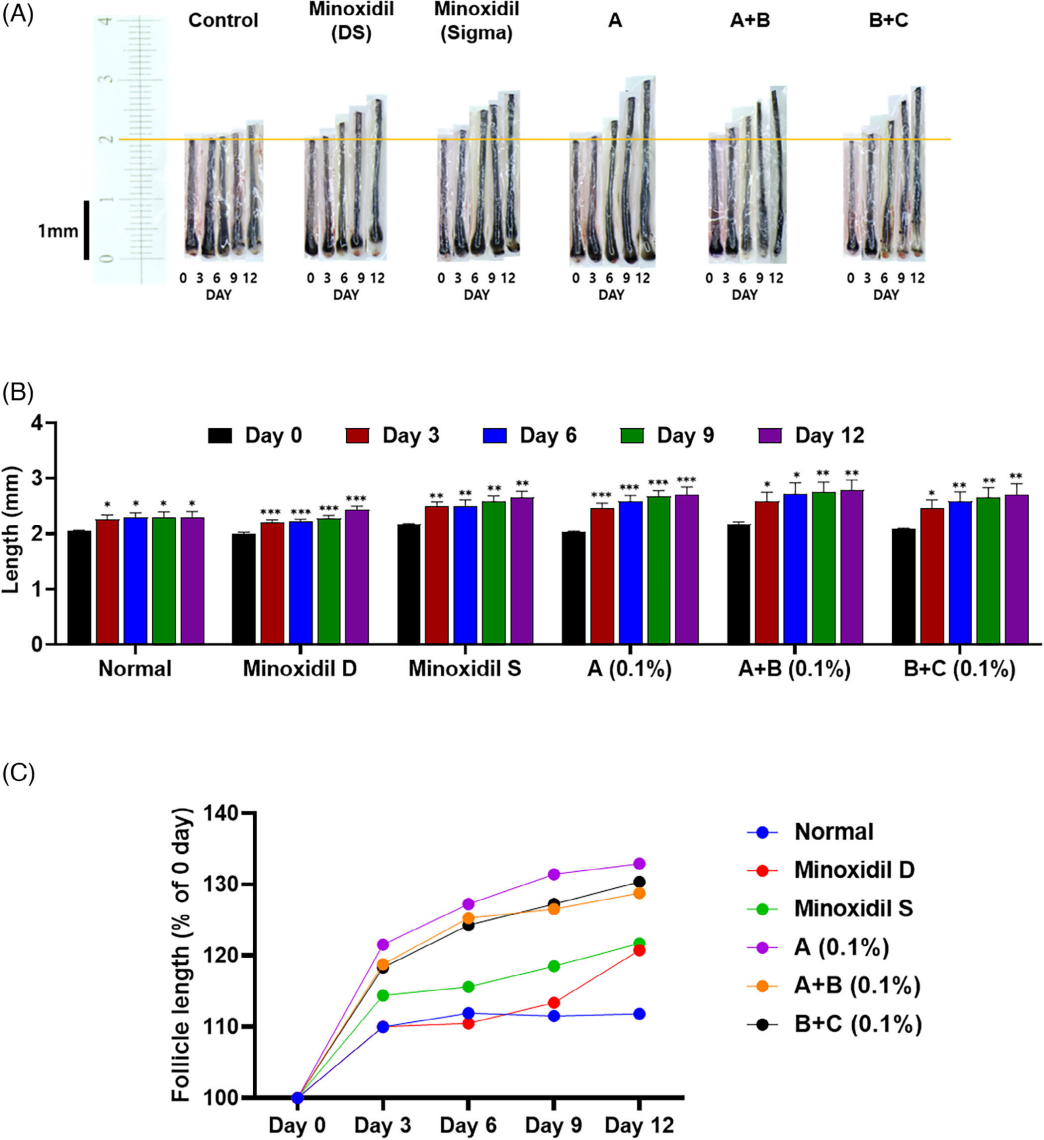
Hair follicle length analysis of test group 1. (A) Visualization of hair length measurement under different treatments (*N* = 8). (B) Quantitative of hair length in a column graph. (C) Quantitative of hair length in a line graph. Group A: Bioscalp 0.1%, group A + B: Bioscalp 0.1% + Ultra Exo Booster 0.1%, group B + C: Ultra Exo Booster + Ultra S Line Plus (C&O) 0.1%.

**FIGURE 2:**
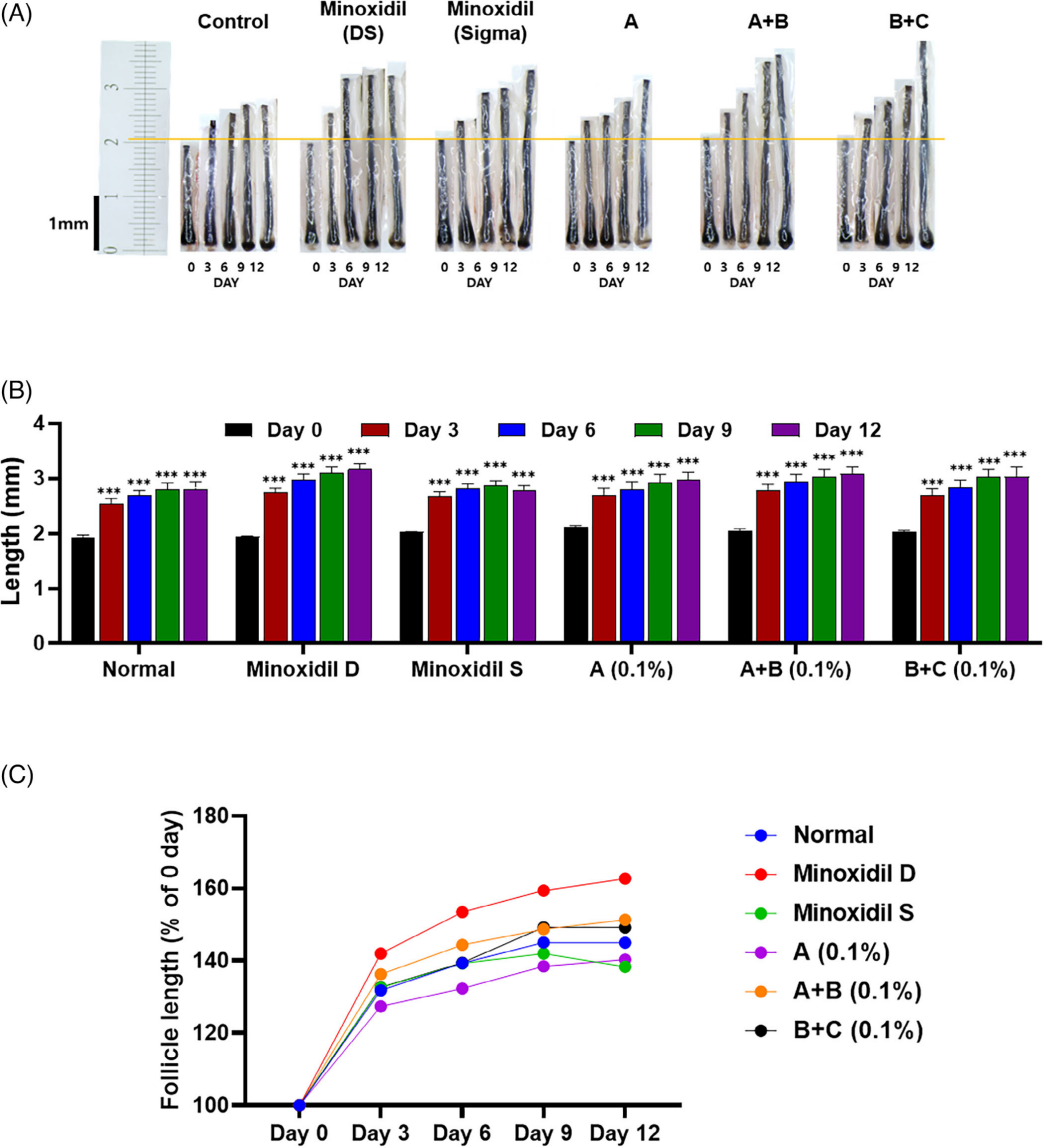
Hair follicle length analysis of test group 2. (A) Visualization of hair length measurement under different treatments (*N* = 8). (B) Quantitative of hair length in a column graph. (C) Quantitative of hair length in a line graph. Group A: Bioscalp 0.1%, group A + B: Bioscalp 0.1% + Ultra Exo Booster 0.1%, group B + C: Ultra Exo Booster + Ultra S Line Plus (C&O) 0.1%.

**FIGURE 3:**
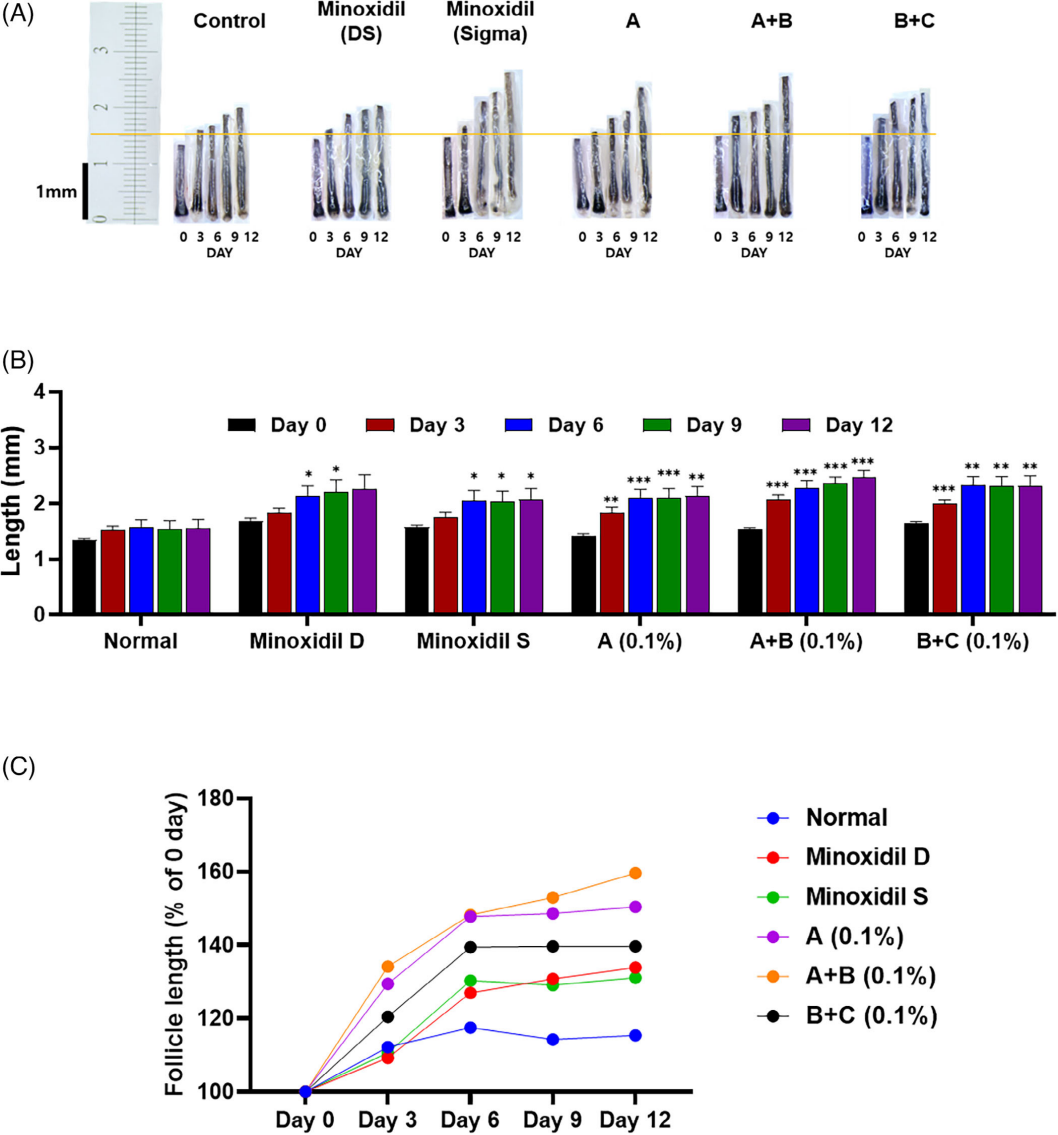
Hair follicle length analysis of test group 3. (A) Visualization of hair length measurement under different treatments (*N* = 8). (B) Quantitative of hair length in a column graph. (C) Quantitative of hair length in a line graph. Group A: Bioscalp 0.1%, group A + B: Bioscalp 0.1% + Ultra Exo Booster 0.1%, group B + C: Ultra Exo Booster + Ultra S Line Plus (C&O) 0.1%.

To evaluate the efficacy of the testing products in stimulating hair growth, testing products from companies were cultured at a concentration of 0.1% for 0, 3, 6, 9, and 12 days. Subsequently, visualization of hair length was conducted by photographing under a dissecting microscope, as shown in Figures 1A–3A.

The quantitative analysis of the first trial exhibited an increment from 2.01 to 2.26 mm (9.7%) after 3 days of hair under an ex vivo typical environment; after that, the hair length almost remained after 12 days (2.26, 2.30, 2.29, and 2.30 mm) (Figure 1B). Sigma minoxidil‐treated hairs interpreted significant changes in hair length between Day 0 and Day 3 and slightly increased the length from Days 6, 9, and 12 from 2.17 to 2.49, 2.51, 2.58, and 2.65 mm (respectively 14.74%, 15.67%, 18.8%, and 22.12% compared to Day 0) as shown in Figure 1B. Additionally, the result of Dongsung minoxidil‐treated hair was almost similar in increasing hair length after 12 days of treatment, with approximately a 9.95% increment from Day 0 to Day 3. On the contrary, test group A with bioscalp product added to 2.59 mm after 3‐day treatment from 2.18 mm, equal to 18.80%, and inclined to increase gradually till 12 days of treatment. However, on Day 12, the length remarkably increased by 32.1%.

To evaluate the functions of exosome‐based products in stimulating hair growth, bioscalp was combined with Ultra Exo Booster, named A+B. The hair length in the group demonstrated a noticeable increase after 3 days of culture (2.18 to 2.59 mm), representing an 18.78% increment (Figure 1B,C), and continuously supported hair growth after 12 days with an increase in 28.44% compared to the first day. In addition, two combined exosome‐based products, Ultra Exo Booster and Ultra S Line Plus, were utilized as a treatment to examine hair growth rate. Initially, the alteration in hair length reached 2.46 from 2.08 mm, which described 18.31% after 3 days of culture. Noticeably, the increment in hair length on Day 12 added up to 30.34% (from 2.08 to 2.71 mm), as depicted in Figure 1B,C.

When initially cultured in 3 days, it could be observed that the hair length in group A (scalp) elevated more than that of other groups, depicting a 21.55% hair growth rate. Comparing the hair growth rate of six groups in percentages in 12 days of culture, test group A manifested the highest value of 32.91% in hair elongation, showing competitiveness to industrial products. Then, exosome‐based test groups B+C and A+B demonstrated 2nd and 3rd place in hair growth stimulation, which showed very close values to A, 30.34%, and 28.78%, respectively.

The quantitative analysis of the second trial described an almost similar trend in hair elongation compared to the first trial, illustrating persistent hair growth in every group after 12 days of culture. As shown in Figure 2, under an ex vivo standard environment without any treatment, it could be visually observed that the hair in the normal group grew gradually in 12 days with an initial length of 1.93 to 2.80 mm, equal to 44.98% (Figure 2B,C). However, in this trial, hairs treated with minoxidil from Dongsung exhibited a more significant growth with a change of 62.69% (1.94 to 3.16 mm) compared to the Sigma‐treated group after 12 days with only 38.27% (2.02 to 2.79 mm) as shown in Figure 2B,C. Nevertheless, group A showed a less competitive growth rate (40.29%) compared to both the normal (44.98%) and Dongsung minoxidil‐treated group (62.69), though the change value was significantly escalated after 12 culture days compared to Day 0. In contrast, group A+B depicted a noticeable escalation in hair length after 3 days of culture from 2.03 to 2.78 mm, equal to 36.22%, and culminated in 3.08 mm (51.28%), occupying the 2nd place after Dongsung minoxidil. Subsequently, the combination of exosome‐based products, group B+C, resided the 3rd place, representing a continuously remarkable progression rate starting from Day 0 to Day 3 and 12 from 2.03 mm to 2.69 and 3.03, amounting to 32.66% and 49.17% (Figure 2C).

Illustrated in Figure 3, the non‐treated group of trial 3 exhibited a fluctuation in hair growth that depicted a modest progressive value in hair elongation, approximately 12.15% from 1.35 to 1.51 mm in 3 days, then inclined to decrease to 14%–15% after reaching a peak at Day 6 (17.49%). Conversely, the steady augmentation of the hairs cultured with Dongsung minoxidil extended considerably after 12 culture days with an extension rate of 33.93% (1.84 to 2.25 mm), which could be observed in Figure 3B,C. Likewise, Sigma minoxidil treated group described an approximately comparable growing pattern of 31.10% in extension rate after 12 days. Impressively, when cultured with test group A, an outstanding result revealed an increment in hair length by 50.4%, as shown in Figure 3B,C. Besides, a considerable value in the progression of hair growth after treatment with group A+B unveiled a gain of 59.64% in elongation represented a progression from 1.54 to 2.46 mm, inhabiting the most competitive product among all groups. Apart from that, group B+C also described a noticeable elongation after 3 days with a 20.41% (1.65 to 1.99 mm) increment and summited at Day 6 (2.32 mm) and maintained the value at around 39.65% from Days 6 to 12. Though the extension rate was significant in group B+C, groups A and A+B manifested a more intense hair elongation at age.

### 2.2 Hair bulb diameter analysis

**FIGURE 4:**
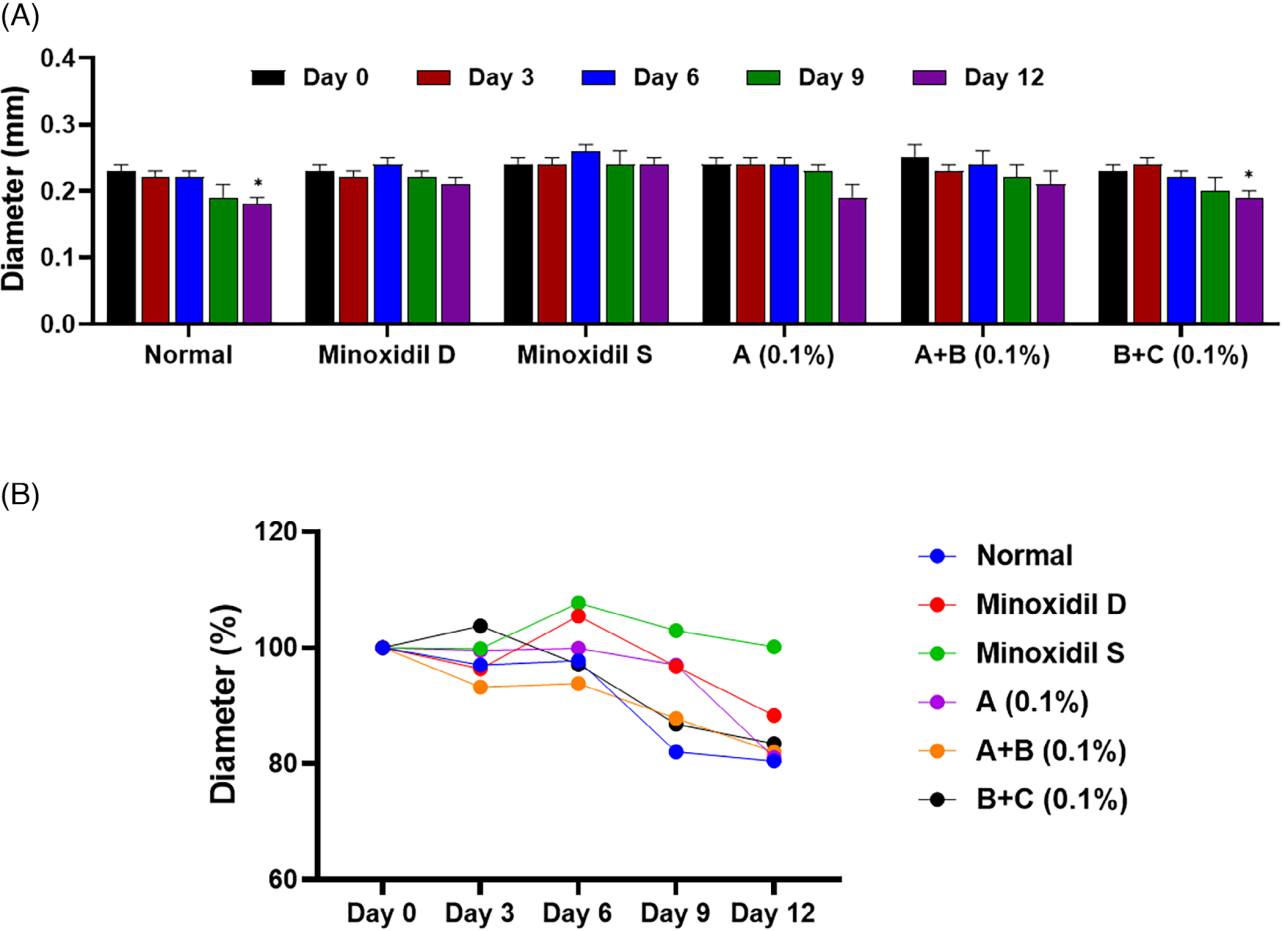
Hair bulb analysis of test group 1. (A) Quantitative of hair bulb diameter in a column graph. (B) Quantitative of hair bulb diameter in a line graph. Group A: Bioscalp 0.1%, group A + B: Bioscalp 0.1% + Ultra Exo Booster 0.1%, group B + C: Ultra Exo Booster + Ultra S Line Plus (C&O) 0.1%.

**FIGURE 5:**
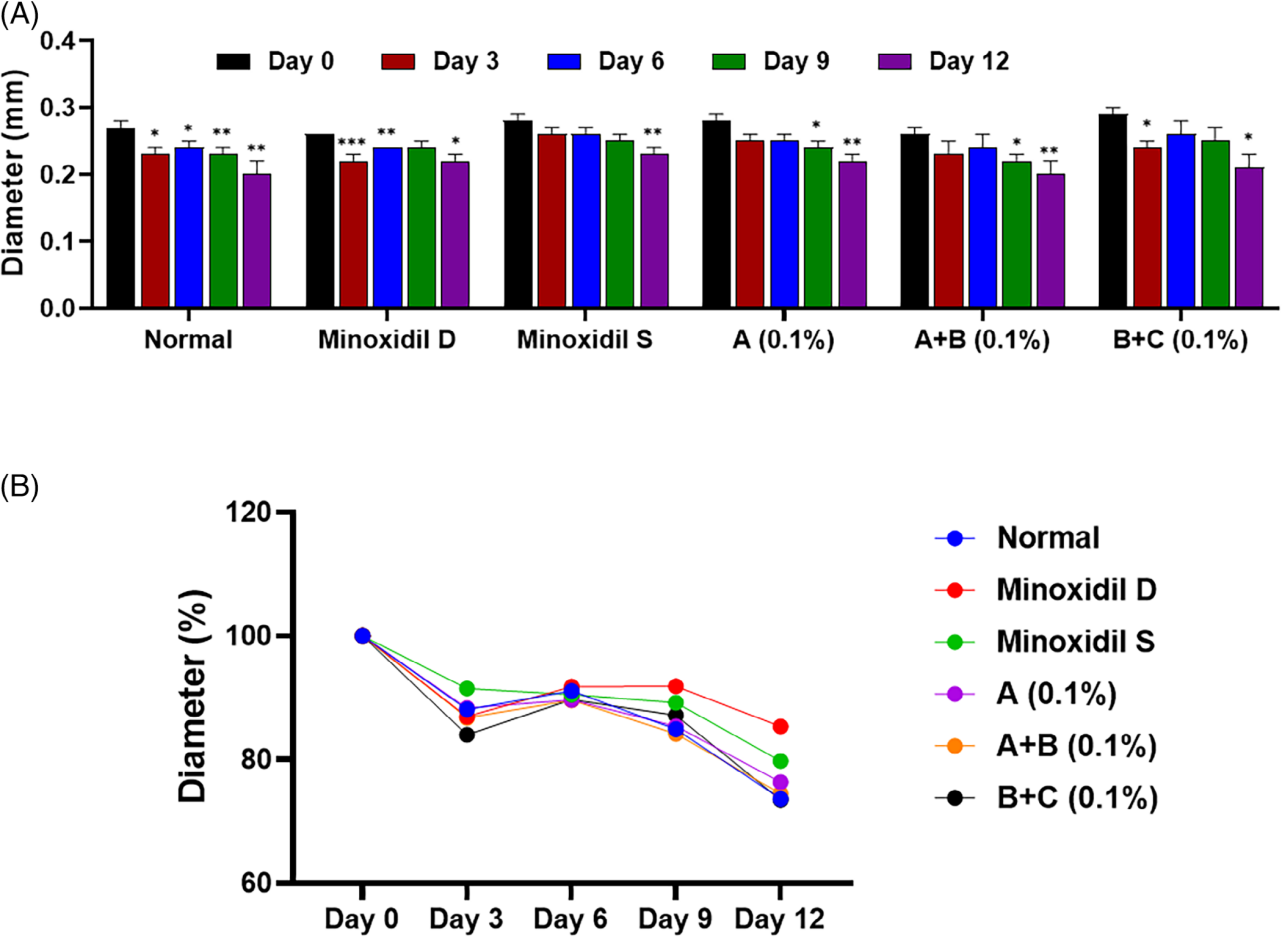
Hair bulb analysis of test group 2. (A) Quantitative of hair bulb diameter in a column graph. (B) Quantitative of hair bulb diameter in a line graph. Group A: Bioscalp 0.1%, group A + B: Bioscalp 0.1% + Ultra Exo Booster 0.1%, group B + C: Ultra Exo Booster + Ultra S Line Plus (C&O) 0.1%.

**FIGURE 6:**
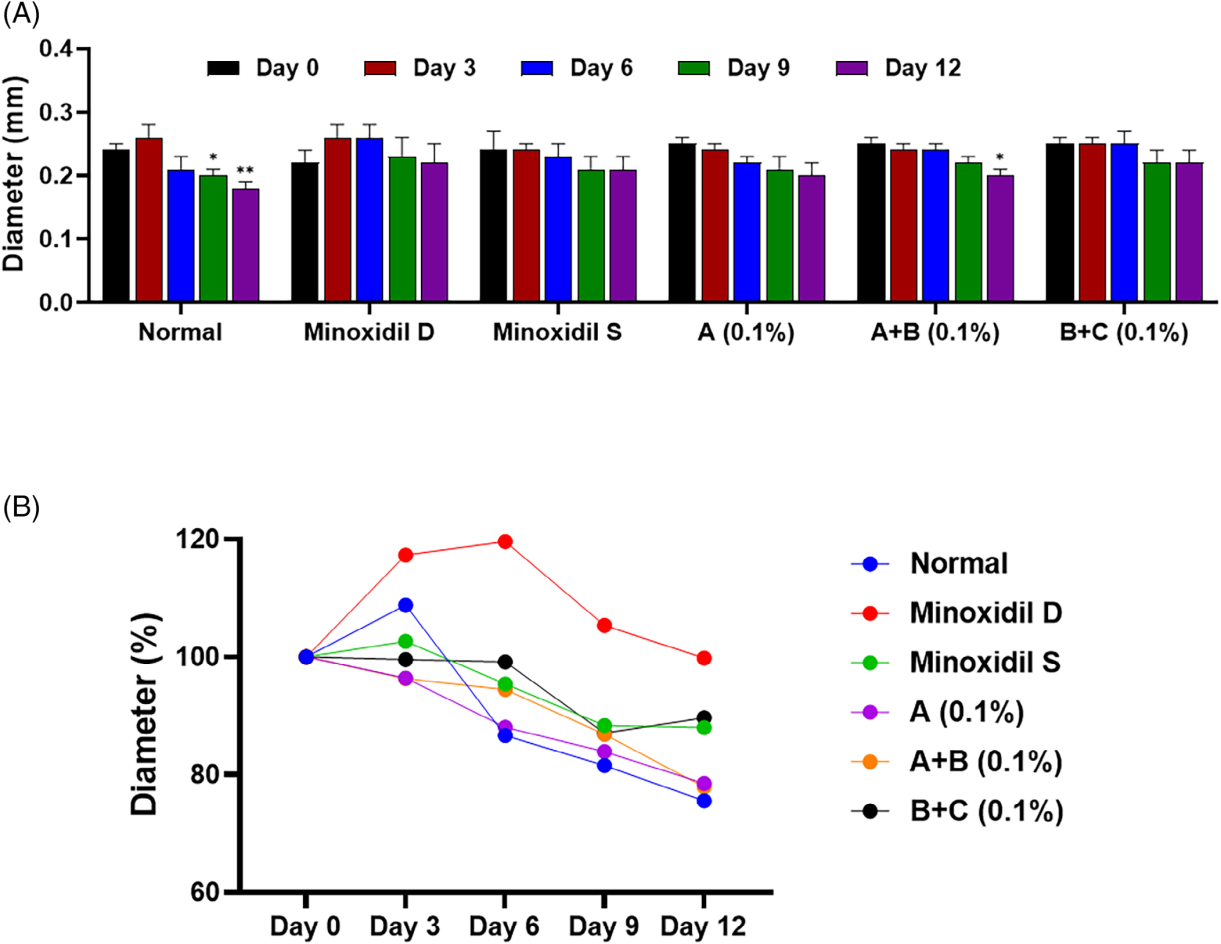
Hair bulb analysis of test group 3. (A) Quantitative of hair bulb diameter in a column graph. (B) Quantitative of hair bulb diameter in a line graph. Group A: Bioscalp 0.1%, group A + B: Bioscalp 0.1% + Ultra Exo Booster 0.1%, group B + C: Ultra Exo Booster + Ultra S Line Plus (C&O) 0.1%.

Hair bulbs, of which living cells are accountable for hair production, including the actively dividing matrix cells, interfere with the process where the process of hair growth initiates. Therefore, we conducted the experiment to evaluate the effectiveness of testing products to sustain the diameter of hair bulbs in ex vivo environments (Figures 4, 5, 6).

Following the evaluation of hair bulb diameters in trial 1, it was observed that after 12 days of hair culture under a standard ex vivo environment, the hair bulbs in the non‐treated group experienced a noticeable reduction of 19.54% in diameter, as shown in Figure 4A. The Sigma minoxidil‐treated group presented a sustainable value in hair bulb diameter in 12 days, while the diameter values of the one treated by Dongsung minoxidil resulted in a decrease of 11.69% compared to Day 0. In contrast, test group A exhibited a significant decrease of 18.94% during the same period. Notably, test groups A+B and B+C illustrated a resemblance trend in the data, which illustrated a decline in 17.97% and 16.54%, respectively, after almost maintaining the hair bulb diameter in 9 days (Figure 4A,B). Although no statistically significant distinctions emerged when directly comparing the groups, an analysis relative to the untreated group suggested a tendency for a lesser change rate in hair bulb diameter within the test group, indicating maintenance of hair bulb diameter compared to the positive control groups.

In trial 2, the untreated group showed a substantially decreased hair root diameter. Reductions of 11.89%, 8.87%, 15.09%, and 26.32% in hair bulb diameter were observed at 3, 6, 9, and 12 days of hair culture, respectively, when compared to Day 0 as shown in Figure 5A,B. Group A displayed notable reductions in hair root diameter, recording decreases of 14.56% and 23.63% on Days 9 and 12, respectively. Test Group A+B demonstrated a significant decrease in hair root diameter, with declines of 15.82% and 28.50% at 9 and 12 days, while Test Group B+C exhibited significant decreases of 15.97% and 26.48% at 3 and 12 days, respectively.

During trial 3, the untreated group experienced a noteworthy decrease in hair bulb diameter, recording 18.41% and 24.42% reductions on the 9th and 12th days of hair culture (Figure 6A,B). In this trial, both the minoxidil groups tended to preserve hair bulb size after 12 days, which depicted a minor change in hair bulb diameter with 0.17% and 11.99%, respectively. Conversely, Test Group A exhibited a significant decline of 22.07%, specifically noted on the 12th day. Besides the marked decrease of test group A+B after 12 days of culture with a decrease rate of 22.07%, the values in test group B+C resulted in ostensible maintenance in hair bulb diameter with a slight change rate of 10.32%. Though direct comparisons between the testing and control groups did not reveal statistically significant differences, the data from the non‐treated group hinted at a propensity for a minimal change rate in hair bulb diameter within the test group. The achieved results implied the preservation of hair bulb diameter of the testing group, especially in the exosome‐based product group, in contrast to the control groups.

### 2.3 Hair growth cycle analysis: Anagen and catagen evaluation

**FIGURE 7:**
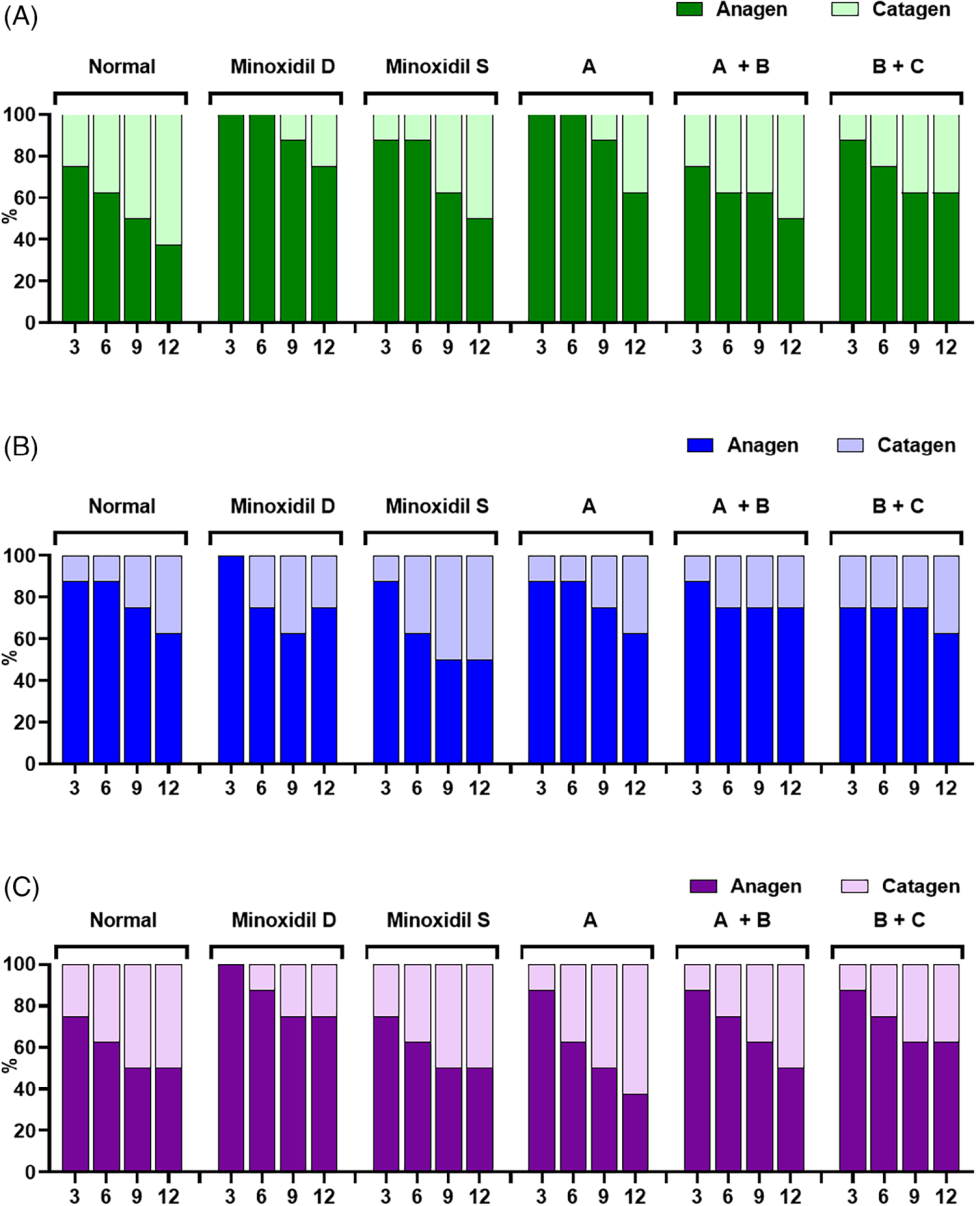
Hair bulb analysis of test group 3. (A) Quantitative of hair bulb diameter in a column graph. (B) Quantitative of hair bulb diameter in a line graph. Group A: Bioscalp 0.1%, group A + B: Bioscalp 0.1% + Ultra Exo Booster 0.1%, group B + C: Ultra Exo Booster + Ultra S Line Plus (C&O) 0.1%.

In the context of analyzing the hair follicle growth cycle, within the untreated cohort, the first trial showed a progressive increase in the percentage of hair follicles transitioning to the catagen stage, reaching 25.00%, 37.50%, 50.00%, and 62.50% on the 3rd, 6th, 9th, and 12th day of ex vivo hair culture, respectively (Figure 7A). In contrast, a subtle transition to catagen of Dongsung minoxidil‐treated groups was observed at 25% after 12 days. Besides, Sigma one described a higher transition status, which showed 50% of catagen within the same periods, while in Test Group A, the proportion of hair follicles progressing to the catagen stage was lower with 12.50% and 37.50% on the 9th and 12th day of hair culture, respectively. Test Group A+B exhibited percentages of 25.00%, 37.50%, 37.50%, and 50.00% for the catagen stage at 3, 6, 9, and 12 days of hair culture, respectively. Similarly, Test Group B+C displayed progression to the catagen stage at 3 and 6 days, with 12.50%, 25.00%, 37.50%, and 37.50% at Days 1, 9, and 12. Upon inter‐group comparison, Test Groups A, A+B, and B+C manifested a tendency to exhibit lower percentages of hair follicles progressing to the catagen stage, registering at 37.50%, 50.00%, and 37.50%, respectively, on the 12th day, in contrast to the non‐treated group.

It was indicated in the second trial that the untreated group exhibited a progression to the catagen stage, with proportions of 12.50%, 12.50%, 25.00%, and 37.50% on the 3rd, 6th, 9th, and 12th days of hair follicle culture, respectively (Figure 7B). Similarly, in test group A, the proportions to catagen transition were 12.50%, 12.50%, 25.00%, and 37.50% within the same period. Test group A+B displayed proportions of 12.50%, 25.00%, 25.00%, and 25.00% at 3, 6, 9, and 12 days while test group B+C had a more significant percentages of 25.00%, 25.00%, 25.00%, and 37.50% for the same time points. In inter‐group comparisons, test group A+B exhibited a lower tendency in the proportion of hair follicles progressing to the catagen stage, specifically at 25.00% after 12 days of culture, compared to the untreated group.

The third trial illustrated that within the untreated group, the percentage of hair follicles transitioning to the catagen stage stood at 25.00%, 37.50%, 50.00%, and 50.00% on the 3rd, 6th, 9th, and 12th days of hair follicle culture, respectively (Figure 7C). Conversely, in test group A, the corresponding proportions were 12.50%, 37.50%, 50.00%, and 62.50%. Test group B+C exhibited progression rates of 12.50%, 25.00%, 37.50%, and 50.00% at 3, 6, 9, and 12 days, while test group B+C indicated advancement to the catagen stage at 3 and 6 days. On Days 1, 9, and 12, the percentages for test group 3 were 12.50%, 25.00%, 37.50%, and 37.50%, respectively. In the comparative analysis between groups, Test Group 3 demonstrated a reduced inclination in the proportion of hair follicles entering the catagen stage, specifically registering at 37.50% on the 12th day, as opposed to the untreated group.

## 3 DISCUSSION

The hair follicle undergoes a three‐phase turnover process, which consists of anagen (proliferation), catagen (involution), and telogen (resting) stages.^23^ A hair follicle is structurally comprised of various cell layers, including the outer root sheath, inner root sheath, germinal matrix, and dermal papilla cells.^24^, ^25^ When dissected from the scalp, the outer and inner root sheath remain to adhere to the hair shaft. The outer root sheath, constituted of epithelial cells encompassing the hair shaft and maintaining a continuous connection with the epidermal layer of the skin, is considered a potential reservoir of stem cells with therapeutic potential.^26^, ^27^ The sheath assists the hair shaft with structural support and formation by supplying sufficient cell formation.^28^ Lying between the outer root sheath and the hair shaft is the inner root sheath that includes three distinct layers of keratinized cells originating from the hair matrix whose keratin is similar to the type found on the skin surface.^29^ The inner root sheath plays several crucial roles in molding newly developing hair shafts, facilitating proper keratinization, and ensuring the secure attachment of the hair fiber to the scalp.^30^, ^31^ The interaction between the outer and inner root sheath is essential in the hair growth process, which is critical for the appropriate formation and growth of the hair shaft.^32^, ^33^

Bioscalp, a recent product of Ultra V, contains several medicinal herbal extracts such as *Polygonum multiforum* root, *Houttuynia cordata*, *Camellia sinensis* leaf, *Scutellaria baicalensis* root, and *Forsythia suspense* fruit. Precisely, polygonum multiform extract with its anti‐androgenic effect was elucidated to stimulate the growth of human hair follicle cells in vivo by elongating the anagen phase.^34^, ^35^, ^36^ *Houttuynia cordata* (HC) is acknowledged for its rich content of natural polysaccharides, making it a valuable source in traditional medicine for immune stimulation, anti‐inflammatory, and anti‐bacterial activities.^19^, ^37^ Though the mechanism of how HC can promote hair growth, research has been conducted to prove that it stimulates hair follicle cell proliferation in vivo and in the C57BL/6 mouse model by enhancing growth factors and metabolism in cells.^38^, ^39^ *Camellia sinensis* leaf, or black tea, which comprises a high concentration of EGCG, an antioxidant compound, has been applied in cosmetic and skin biochemistry.^40^, ^41^ In the inner layers of the skin, the extract demonstrates notable protective properties against lipoxygenase, metalloproteinase, hyaluronidase, and collagenase enzymes, attributing to preventing skin aging.^40^ As in our research, the product Bioscalp with listed ingredients illustrated a remarkable impact on hair elongation and postponed catagen progress of hair follicles in 12 days of ex vivo culture. It is hypothesized that the keratinized cells located in the inner root sheath and epithelial cells in the outer root sheath were proliferated and cultivated under an ideal environment with several supportive conditions from herbal extracted enabled consolidation of the existing molding structures, leading to hair elongation and less catagen progression than the normal group.

Ultra Exo Booster is a product for mesotherapy treatment of skin problems from Ultra V in which exosomes are applied in a mixture originating from cord blood stem cells that contain a lot of growth factors that help skin regeneration. In dermatology, exosome cultivates skin rejuvenation, increases type I procollagen, and reduces MMP‐1, stimulating collagen recovery.^42^, ^43^, ^44^ The rejuvenation solution Ultra Exo Booster was reported with a formula of 89 ingredients: Besides the outstanding Exosome ingredient, Exo Booster also contains hyaluronic acid 1.5%, PDRN, 13 vitamins, 20 amino acids, 5 minerals, 4 peptides, 6 herbs,… Polydeoxyribonucleotides (PDRN) promote the growth and activity of fibroblasts, while exosomes act as messengers for skin cells.^45^, ^46^ Additionally, the products incorporate peptides and amino acids, contributing to the thickening of hair and promoting overall health and maintenance.^47^, ^48^ Since exosomes have been reported to range from 30 to 150 nm, it is effortless for exosome particles to go between cells, promoting osmosis of the product just by external application.^49^ Therefore, Ultra Exo Booster was expected to cultivate hair culture ex vivo. Exosomes have been elaborated to modulate hair growth as an innovative method for hair loss. Hence, a combination of Bioscalp and Ultra Exo Booster was utilized to evaluate and compare the effectiveness in aiding hair growth. As depicted in the research, the results demonstrated a noticeable alteration in hair length after ex vivo culture after 12 days. Though the result in preserving hair bulb diameter and anagen ratio was not as high as the industrial minoxidil groups, it was still significant compared to the non‐treated one, providing a promising treatment for hair loss in the future.

According to the explanation of the company, Ultra S Line C&O by Ultra V is also a mesotherapy‐liquid essence that helps prevent fat accumulation in the chin, abdomen, biceps, and calves, which is capable of boosting lymphatic circulation and facilitating fat loss through natural metabolism and elimination processes. The product contains aesculus hippocastanum extract, Lecithin, AMP, and ATP. Noticeably, lecithin itself was reported to aid in optimizing the necessary protein levels for hair growth and create a protective barrier on the skin and hair by its high concentration of fatty acids, consequently enhancing the texture and appearance of hair by imparting shine and luster.^50^, ^51^ Research has evaluated lecithin‐base nanoparticles for minoxidil delivery and suggested hair growth enhancement via follicular administration using lecithin‐based products.^52^, ^53^ With all the above evidence, the incorporation of exosome‐based products Ultra Exo Booster and Ultra S Line was used to assess the effectiveness of co‐culture of the two fostering products in hair culture. The results of examining hair length implied a remarkable potential for boosting hair growth. Moreover, the hair bulb diameter measuring and catagen analysis data reflect the product's effectiveness for hair growth. The evidence is that compared with the commercial product group, this product demonstrated better hair root preservation and sustained hair growth longer during the 12‐day culture under the ex vivo environment.

## 4 CONCLUSION

Our research is centered on evaluating innovative approaches to overcome hair loss issues. The study explores advanced solutions using exosome‐based nanocarrier technology and medicinal herbal encapsulated formulae to stimulate hair growth under an ex vivo environment. New formulations, including Bioscalp and Ultra Exo Booster, are introduced whose effectiveness in promoting hair elongation and preserving hair bulb integrity demonstrated a potent effectiveness. Bioscalp, combined with Ultra Exo Booster and Ultra S Line Plus, shows significant hair elongation. The study concludes that these formulations, particularly the combination of Bioscalp, Ultra Exo Booster, and Ultra S Line Plus, exhibit significant efficacy in fostering hair growth, preserving hair bulb integrity, and minimizing the transition to the catagen stage. These findings suggest promising alternative treatments for hair loss with improved outcomes compared to current options.

## 5 METHODS

This study has been approved and confirmed by the Korean Skin Research Center Ethics Committee, H&Bio Corporation, Seongnam, Korea. All research in this manuscript was performed in accordance with relevant guidelines.

### 5.1 Test materials and test conditions

1. Untreated group (Normal).
2. Positive control 1: Minoxidil (Dongsung) 0.20 ppm.
3. Positive control 2: Minoxidil (Sigma Aldrich) 0.20 ppm (=1 µM).
4. Test group A: Bioscalp 0.1%.
5. Test group A+B: Bioscalp + Ultra Exo Booster 0.1%.
6. Test group B+C: Ultra Exo Booster + Ultra S Line Plus (C&O) 0.1%.

For test group 2, use 5 mL of Bioscalp by dissolving it in Ultra Exo Booster using a syringe. For test group 3, use 5 mL of Ultra S Line Plus (C&O) by dissolving it in Ultra Exo Booster using a syringe.

### 5.2 Hair follicle isolation and culture

The scalp tissues were washed twice with PBS (Phosphate‐Buffered Saline) and placed in a sterilized Petri dish. Separate hair follicles under a dissecting microscope based on the dermis. Remove as much fat tissue around the separated hair follicle tissue as possible and cut it to a certain length based on the dermal papilla (DP). Hair follicles are cultured in William's E medium with 2 mM L‐glutamine, 10 µg/mL insulin, 10 ng/mL hydrocortisone, and 1% antibiotics in an incubator at 37°C and 5% CO2. For hair follicle organ culture, untreated group, positive control group, Minoxidil (Dongsung) 0.20 ppm and Minoxidil (Sigma) 0.20 ppm, test group 1 (Bioscalp 0.1%), test group 2 (Bioscalp + Ultra Exo Booster 0.1%) And in test group 3 (Ultra Exo Booster + Ultra S Line Plus (C&O) 0.1%), hair follicles were cultured in medium treated with each substance (1 mL/24‐well plate) for up to 12 days. On Days 0, 3, 6, 9, and 12 of culture, hair follicles are observed, and hair follicle length and hair root diameter are measured through photography.

### 5.3 Measurement of hair follicle length and root diameter (hair bulb)

Hair follicles were photographed using a dissecting microscope (SZ51, OLYMPUS, Japan) and ToupLite software (ToupTek, China) at 0, 3, 6, 9, and 12 days of culture, and Image J (The National Institutes of Health, USA) program was used to measure hair follicle length and hair root diameter.

### 5.4 Hair follicle growth cycle evaluation

Hair follicles were photographed using a dissecting microscope (SZ51, OLYMPUS, Japan) and ToupLite software (ToupTek, China) on Days 0, 3, 6, 9, and 12 of culture, and the hair root portion of the hair follicle structure was analyzed. It mainly distinguishes between the growth period (Anagen) and the regression period (Catagen).

### 5.5 Statistical analysis

1. All calculated data are verified for statistical significance using SPSS Package Program version 20 (IBM, USA).
2. The normality of the data is verified through the Shapiro‐Wilk test and kurtosis&skewness.
3. Analysis of Variance (Repeated Measures ANOVA) is used to compare before and after, and between groups at each time point (*p* < 0.05).
4. The hair follicle length change rate, hair root diameter change rate, and hair follicle growth cycle rate are calculated as follows.

## AUTHOR CONTRIBUTIONS

Sun Young Nam and Chan Yeong Heo conceptualized the study and designed it together with Hyunjee Kim and Nguyen Ngan Giang. Han Jin Kwon, Jung Ryul Ham, Won Ku Lee, and Yeon Ju Gu collected and prepared the samples. Data collection and analysis were performed by Pham Ngoc Chien, Hyunjee Kim, Linh Thi Thuy Le, and Nguyen Ngan Giang, with Shu Yi Zhou, Pham Thi Nga, Trinh Thi Thuy Tien, and Xin Rui Zhang in a supporting role. The first draft of the manuscript was written by Nguyen Ngan Giang. All authors commented and confirmed the final version of the manuscript. All authors read and approved the arrangement.

## CONFLICT OF INTEREST STATEMENT

The authors declare that they have no competing financial interests.

## ETHICS STATEMENT

This study has been approved by the Ethics Committee of the Korean Skin Research Center, H&Bio Corporation, Seongnam, South Korea. Scalp tissue from Korean adults aged between 20 and 80 without skin disease was used in the study with IRB approval from the KINS Korean Institute of Nonclinical Study with IRB code HBABN01‐220510‐BR‐E0116‐01. Informed consent to publicly use the participants’ information was obtained from all participants.

## Data Availability

The data that support the findings of this study are available from the corresponding author upon reasonable request.
